# Models for projecting supply and demand for nurses in Israel

**DOI:** 10.1186/s13584-015-0043-6

**Published:** 2015-12-15

**Authors:** Nurit Nirel, Orli Grinstien-Cohen, Yonatan Eyal, Hadar Samuel, Assaf Ben-Shoham

**Affiliations:** Smokler Center for Health Policy Research, Myers-JDC-Brookdale Institute, JDC Hill, POB 3886, Jerusalem, 91037 Israel; Faculty of Health Sciences, Recanati School for Community Health Professions, Ben-Gurion University of the Negev, Beer-Sheva, 84105 Israel

## Abstract

**Background:**

Concern is growing over serious shortages in the nursing workforce and imbalance between supply and demand. Projections indicate that the demand for the nursing workforce will increase due to the aging population and an increase of the percentage of elderly people requiring assistance.

**Study goals:**

To examine the expected balance between supply and several demand projections for nurses in Israel in order to contribute to planning the nursing workforce.

**Methods:**

1. Open interviews with key figures in the healthcare and nursing care systems; 2. Examination of supply and demand for nurses; 3. Examination of the balance between supply and demand projections.

**Main findings:**

A considerable gap was found between the supply and demand projections for registered nurses, which will increase over time according to each of the demand projection models up to 2030. All of the models indicate that the projected shortage will be significantly affected by the age at which the nurses retire. Models based on a fixed ratio of nurses or infrastructure (beds, positions) per population show a particularly great gap between demand and supply. However, a more conservative model (based on hospital utilization), that takes the system's infrastructures and limitations, as well as the growing population and changes in its composition into account, without an increase in the direct ratio of the number of nurses, also predict a significant shortage of nurses within 20 years.

**Conclusions:**

The gaps between the demand and supply projections indicate the need to augment the workforce in addition to the steps currently taken to recruit nursing staff and increase the number of training institutions for nurses. The relatively simple supply prediction models, which are based on available sources of information that can be easily revised, will make it possible to monitor and update projections regularly over time. The models developed in this study should help the process of long-term strategic planning for the number of nurses in Israel.

## Background

Faced with an anticipated gap between supply and demand, over the past decade, the Western world has become increasingly concerned with the nursing workforce^1^ [1–4]. This concern is based mainly on projections indicating that the demand for the nursing workforce will increase due to the growing aging population, at a time when the nursing profession is having difficulty attracting new staff.

The planning of the healthcare workforce is closely based on health policy and the changes in society as a whole (e.g., demographic changes and the disparities in the health status of population groups), as well as changes in the health system, and in the professions examined. With regard to nursing, three systems in Israel have undergone changes that affect demand:

1. Geriatric hospitals, whose share of the hospital system has increased dramatically and where the treatment of patients is chiefly nursing care
2. General hospitals where, due to a decline in the average hospital stay and the multiple medical treatments in the community (thanks to medical developments and new technology), the percentage of severely ill patients, older patients, and more complex patients has increased;
3. Health services in the community, which are now responsible for many functions for which nurses have to be trained to work independently, particularly when treating older patients with chronic illnesses.

The above changes have implications for both the size and professional mix within the nursing workforce [6–8].

Accepted workforce planning models examine both the supply and demand for a given profession. "Supply" in terms of workforce planning indicates the size and characteristics of the available workforce at a given point in time [9]. This is determined by the currently employed (active) workforce, new members likely to join the workforce by a certain time, and members likely to leave the workforce by a certain time due to retirement, a career change, death, or emigration [5, 10–12].

The terms "demand for workforce" or "workforce requirement" apply to the workforce needed in order to provide health services at a specified level or the desired level. A literature survey of models used to examine the demand for workforce shows them to be on a continuum from basic simple models to more complex models. The professional workforce/population ratio is frequently used to assess the demand for the medical or nursing workforce [13]. Some studies examine the demand for workforce by looking at the actual labor market [14]. Information on the labor market via surveys of employers may provide a good estimate of the shortage or surplus of professional staff and even show the reasons for a shortage [15], mainly in the case of short-term workforce planning. A different approach is setting targets for health service provision and expressing them in terms of the required workforce [12].

Yet another approach, based on utilization of the health services, attempts to assess the future demand according to current consumption characteristics ("profiles of consumption") for each age cohort and sex, based on the anticipated rates for each group in relation to the population [16]. The point of departure for this approach is that the current volume and mix of medical services are a worthy point of departure. Inherent in this is the assumption that health needs, according to age and sex, remain constant and that it is possible to predict demographic characteristics of the population on the basis of current trends.

The approach based on the effective demand for health services is also used to identify the demand for medical or nursing workforce [17]. Models based on this approach assume that the demand for nursing workforce hours are based on the demand for health services, patient behavior, prevalence of illness, and other inputs in the health services, such as the number of beds or hospitalizations. In this context, some define the demand for nurses as the number of nurses that employers are willing to employ taking account of financial considerations, the work environment, the health services or changes in them, such as technological changes, demographic changes, and above all, the rapid increase in the percentage of elderly patients [5, 18]. Accordingly, the demand model used by the Health Resources and Services Administration in the United States combines data from databases with demand prediction equations. The model has two main components: 1. The data and equations for predicting the future demand for the health services; 2. The data and equations for predicting the future level of employment of nurses. The model calculates the anticipated use of health services by combining national patterns of health service utilization with population projections by age and sex. The next stage of the model predicts the future level of employment of nurses, i.e., the number of full-time (or equivalent) nurses for each hospital day, as a function of the current level of employment and projects for future trend (affected, for example, by the average level of severity of patients). The combination of forecast utilization of the health services and the level of employment of nurses gives the projected demand for nurses by framework and year [5].

Future demand can also take account of expert opinion regarding the sources for the required workforce based on the health needs of the population [17, 19]. This approach considers the demand for nurses an epidemiological concept based on specific needs dependent on the age and sex of the population, which that are not dependent on current service utilization. This approach is based on three basic assumptions: 1. All health needs can and must be met; 2. Regarding costs, it is possible to identify and implement efficient methods in order to meet the needs; 3. The resources devoted to healthcare are implemented accordingly as needed.

The models based on the level of service use as well as those based on health needs require sophisticated inputs of information. They are feasible in countries where a large quantity of statistical data has been collected and is available, both at the level of service utilization and the great number of variables that affect the demand for health services. This may explain why until now no demand projections for the workforce have been made in Israel and workforce planning has been based on the supply projections [8, 20, 21].

In Israel, the demand for nursing staff in hospitals (where most of the nursing workforce is employed) is determined largely by the standard nursing staffing positions (nurses and auxiliary staff) per bed, which were fixed in the 1997 collective agreement between the public employers and the nurses' union. Presumably, population growth over time should lead to an increase in the number of beds and hence to the number of positions. However, it has been many years since hospital beds have been added to meet population growth. In the past twenty years, the number of beds in general hospitals has increased by about 21 %, while the population has grown by about 65 % [22]. It should also be noted that since the 1990s, there has been almost no change in the fixed ratio of nursing positions per bed. For this reason, the current staffing positions do not reflect changes in complex morbidity of the patients nor in production functions (technology, medical and nursing professions).

This study sought to examine the demand projections for nurses, taking all the above facts into account. The study goals were to assess the anticipated projections for nurses, to examine the balance between the supply and demand projections, and to understand what this means for planning the nursing workforce in Israel.

## Study method

### 1. In-depth Interviews with Key Personnel in the Health System and Nursing Profession

In order to examine how the hospital and community workforce is determined, a series of in-depth interviews was conducted with key personnel in the health system and nursing profession, among them, the heads of nursing in hospitals and the health plans and senior administrators in the hospitals, health plans, Ministry of Health and the Civil Service Commission.

### 2. Supply projections

**Fig. 1:**
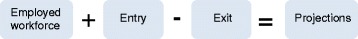
Supply projection model

The supply projections were based on a supply projection model (see below) described by Nirel et al. [21]. The model examined the projected supply of nurses at several points in time based on the current nursing workforce and the future sources for additional workforce versus the forecast numbers of nurses leaving of the profession (leaving the profession, retirement, death, or migration) at the same points in time (Fig. 1).

The supply projections are based on intervals of five years (base year, 2010) and on two scenarios: 1. Nurses remaining in the workforce until age 60; b. Nurses remaining in the workforce until age 65. Both scenarios are based on the following data:

- Existing workforce: The percentage of nurses working in the profession out of all registered nurses of working age, which is 89 % (Nirel et al. [21]), divided by age.
- The projected annual rate of nurses entering the nursing workforce, divided into age cohorts, based on data from the Nursing Administration at the Ministry of Health, and, based on the findings of the said study, the expected percentage of nurses temporarily leaving the workforce and returning to it every year, by age cohort.
- The projections for nurses leaving the workforce (including retirement): The data are based on the results of the survival analyses calculated according to the data in the said study, which presented the likelihood of survival (or quitting) the profession in a period, by age cohort; estimated rate of annual migration of nurses, which is 0.0076 in every age cohort according to data from the Nursing Administration at the Ministry of Health regarding the number of nurses requesting documentation in order to work abroad; calculation of mortality rates, by age group, based on the mortality rates per 1,000 capita for women according to data from the Central Bureau of Statistics (CBS).

### 3. Demand projections for nursing workforce and nurses

The demand projections for nurses were based on three models: One model for total nurses in Israel and two for nurses in hospitals. The demand projections for nurses in the community were added to each of the latter two. The projections were made for 2015, 2020, 2025 and 2030. For the demand projections, we used the population in terms of standard capita according to the capitation formula used for allocating the National Health Insurance Law funds to the health plans. This formula gives differential weights according to age, gender and geographical residence (center or periphery) – variables that affect the consumption of health services.

**Fig. 2:**
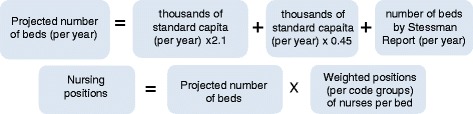
Formula for calculating the demand for nurses according to model B]

**Fig. 3:**
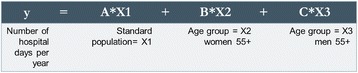
Regression formula for predicting the connection between the population size and composition and the number of hospital days

- Model A: Demand projection for total nurses in Israel by the nurse-to-population ratio (per thousand standard capita): We examined the ratio of nurses per thousand standard capita in 2015, 2020, 2025 and 2030, based on a ratio of 4.8 employed nurses per thousand capita (the average ratio of nurses per thousand capita from 2009-2011).
- Model B: Demand projection for nurses in hospitals by a fixed ratio of number of beds per 1,000 standard population: The quantity of nursing staff in hospitals is determined according to the number of nursing positions per bed and differs according to the type of hospital bed. This standard was fixed for the total nursing workforce per bed, without distinction between nurses and auxiliary staff. The demand projections for the number of nursing positions in hospitals was "taken" from the projected number of total positions in the nursing workforce, based on a mix of nurses and auxiliary staff in the wards. The projections were calculated separately according to the ratio of beds per thousand standard capita for each type of bed:
  - oAccording to a scenario of 2.1 beds per population in general wards in general hospitals, based on Ministry of Health projections for general hospitalization [22].
  - oAccording to a ratio of 0.45 beds per population in psychiatric hospitals, based on an agreement between the Ministry of Finance and the Ministry of Health [23].
  - oLong-term hospitalization (geriatric and rehabilitation beds), according to the number of beds and estimated nursing workforce required to care for the future population of Israel, as determined by a special committee chaired by Prof. Jochanan Stessman (hereinafter, the Stessman Committee), appointed by the director general of the Ministry of Health for this purpose [24]. The following is the formula for calculating the demand for nurses according to this model (Fig. 2):
    The projected number of beds for the group of beds with the same nursing workforce position coefficient per bed (code groups) was calculated by dividing it by the total number of hospital days. The size of the future population was calculated by CBS projections for each one of the years for which we made projections.
    Some of the hospital nursing workforce positions is not directly connected to the number of hospital days or the number of approved beds. For example, the nursing workforce positions in outpatient units, the maternity wards, emergency room and dialysis units and is determined according to the number of service users (such as the number of visits or number of births) we added the calculation of the demand for nurses in these units to the calculation of the demand according to this model.
    In order to calculate the demand projections for the total number of nurses in Israel, we added the demand projections for nurses in the community – in the health plan services and public health services – to this demand model (for calculation formula, see endnote 2).
- Model C: Demand projection for nurses in hospitals by utilization (projected number of hospital days). The demand projections by utilization were conducted in two stages:
  Stage 1: Predicting the association between the size and composition of the population (number of standard population weighted using the capitation formula for groups by age, sex, and geographical area) and the number of hospital days by means of linear regression of data for the years 2001-2010. Altogether, regression analyses were conducted for 11 code groups (groups of departments with the same fixed ratio of nursing positions per bed). The dependent variable in each regression was the total number of annual hospital days in each of the departments included in the code groups in all the hospitals in Israel (general, psychiatric and long-term care) (Fig. 3).
  Stage 2: Nursing workforce projection by utilization. In this stage, we used the regression model to examine the association between the size and composition of the population – age, sex and geographic area (standard capita) – and the number of hospital days in the wards (the product of the first stage) to predict the number of hospital days in 2015, 2020, 2025 and 2030. According to the future number of hospital days, we calculated the number of beds by type of department for each of the years, and according to the ratio of nursing workforce to the number of beds, the demand for nursing workforce positions at the four points in time was estimated.
  To this model, too, we added the demand projections for the outpatient departments and the demand projections for nurses in the community.
- Calculating the demand in terms of the number of nurses instead of in terms of numbers of filled positions: In order to convert the number of full-time positions into the number of nurses required, we multiplied the demand for nursing positions by a conversion coefficient, which was calculated separately for hospitals and the community. The coefficient was calculated according to the average number of weekly hours per nurse, according to distribution of the percentage of nurses by the number of hours in the study of Nirel et al. [21]. The conversion coefficient for the hospitals was 1.156 nurses per position. For the community, it was 1.32 per position.
- Creating a common basis for the supply of nurses in the base year: The demand models are based on data of employment of nurses in hospitals and in the community, and come from different sources. Furthermore, the labor market for nurses includes branches of the economy or places of work for which the data were not available and were not included in the demand projections. Examples include nurses working in industry, in institutions belonging to the Ministry of Social Affairs and Services, and in drug rehabilitation centers in the community. In order to create a common basis for the demand of nurses in the base year, it was decided to treat the supply of employed nurses during that year as the demand for nurses in each of the models, and add the difference in the number of nurses between the projected demand and supply (2.5 % in 2010) in each of the projected points in time.

### 4. Examining the balance between the projected supply and demand for nurses

The balance between the projected supply and demand was examined at intervals of 5 years: 2015, 2020, 2025 and 2030 according to two scenarios: 1. Nurses remaining in the workforce until age 60; 2. Nursing remaining until age 65.

## Results

### Supply projections for nurses in the workforce

#### Nurses joining the workforce

The data on nurses joining the workforce were obtained from the Nursing Administration at the Ministry of Health [25], according to which there were 57,609 registered nurses in Israel at the end of 2010. Of these, 41,495 were aged up to 60; 46,740 were aged up to 65. According to Nirel et al. [21], 89 % of the registered nurses in Israel are working in their profession. It can therefore be assumed that in the base year (2010), there were 36,930 nurses working in the profession (scenario up to age 60) or 41,600 (scenario to age 65).

Efforts have been made for several years to increase the trained nursing workforce. These efforts include a considerable increase in the number of students on academic training tracks in nursing school, the introduction of programs for academic retraining for registered nursing (about 500 recruits per year), and the devising of a 2.5-year nursing training program offering a nursing diploma [25]. Accordingly, some 1,200 nurses from all these qualification programs (academic and diploma) can be expected to enter the workforce each year from 2010 to 2014. Subsequently, we can expect approximately 1,800 licensed nurses to enter the workforce every year.

#### Supply projections

Table 1 presents the supply projections for registered nurses in 2015, based on age cohort. As shown, the number of nurses (up to age 65) working in the nursing profession, which totaled 41,601 in 2010, is expected to decline to 39,700 in 2015 – a decline of 4.5 % at the end of 5 years. Likewise, we have predicted the supply of nurses at intervals of 5 years – 2020, 2025, and 2030 – for nurses remaining in work until 65 and for nurses remaining in work until 60.

**Table 1:** Nurse supply projections for 2015, by age group

Age group	Distribution 2010^a^	After 5 years^b^	Licensed nurses joining during 5 years^c^	Retirement and exit from workforce during 5 years^d^	Emigration during 5 years^e^	Death during 5 years^f^	Returning to workforce during 5 years^g^	Supply projection end of 2015^h^
Total	41,601	36,930	6,000	1,437	1,581	350	138	39,701
24-29	4,502		3,872	450	171	5	38	3,285
30-34	5,639	4,502	573	451	214	8	50	4,450
35-39	5,639	5,639	573	169	214	14	25	5,839
40-44	5,639	5,639	573	56	214	23	7	5,926
45-49	5,420	5,639	409	163	206	35	12	5,656
50-54	5,420	5,420		54	206	57	3	5,106
55-59	4,671	5,420		47	178	82	2	5,116
60-65	4,671	4,671		47	178	126	2	4,323

^a^The number of employed nurses up to age 65 at the end of 2010. A survey conducted in 2008 [21] found that 89 % of the nurses registered at the Ministry of Health were working in the profession. Accordingly, the calculation is based on 89 % of the number of registered nurses.

^b^The distribution of nurses employed by age group after 5 years (each group was moved in full to the following age group)

^c^Distribution of the expected entry of licensed nurses by age group based on Ministry of Health data on the average number of new nurses per year between 2010 and 2014, divided into age groups (1,200 new nurses per year)

^d^The calculation for the exit rate (retirement, stopping work as registered nurse) was based on the outcomes of the survival analyses by age, which was conducted in a study on the supply of nurses. The figures were calculated as the product of the rate of those leaving times the number of nurses who were in the age group at the start of the period.

^e^The emigration rate of 0.0076 in each of the age groups was multiplied by 5 in order to reflect emigration over 5 years.

^f^The product of the mortality rate per thousand capita (women) in the age group (CBS data) times the number of nurses in that group multiplied by 5 years and divided by 1,000.

^g^The percentage of nurses likely to return to the profession after a temporary exit from work as registered nurses in every age group multiplied by the number of nurses in each age group who left in the course of 5 years.

^h^The total number of nurses at the end of the period, the number of new nurses and the number of those likely to return to work (columns 3, 4, and 7 respectively) less the total of the columns representing exit from the work force – quitting, emigration and death (5, 6, and 7, respectively).

Table 2 presents the supply projections for nurses remaining in the workforce until age 65, by dates. It shows an increase in the total projected supply of nurses in the workforce from 41,600 nurses working in the profession in 2010 to 43,680 nurses working in the profession 20 years later (in 2030) – an increase of 5 % by the end of that time. The supply projections for nurses in the workforce until 60 indicates an increase from 36,930 in the base year to 38,960 nurses in 2030, an increase of altogether 5 %.

**Table 2:** Nurse supply projections to age 65, in five-year periods

Base year	2010	2015	2020	2025
Total nurses employed in base year	41,601	39,701	41,410	42,436
After 5 years (with no new recruits)	36,930	35,378	36,616	38,020
Licensed nurses joining during 5 years	6,000	9,000	9,000	9,000
Retirement and exit from workforce during 5 years	1,437	1,234	1,377	1,512
Emigration during 5 years	1,581	1,509	1,574	1,613
Death during 5 years	350	345	358	358
Returning to workforce during 5 years	138	119	128	142
Period end year	2015	2020	2025	2030
Nurse supply projects at end of period	39,701	41,410	42,436	43,680

### Demand projections for nurses in the workforce

Table 3 presents an integrated summary of the demand projections (hospitals –inpatient and outpatient departments – and the community) for nurses in each of the following years: 2015, 2020, 2025 and 2030. It also presents a model for the demand projections according to the ratio of nurses in the total population for the same years. The table shows that all the models anticipate a significant increase in the demand for nurses within 20 years. The demand projections for each of the models are as follows:

**Table 3:** Nurse demand projections, by demand models (selected scenarios)

	2015			2020			2025			2030		
Demand projections for registered nurses	(1) Ratio of 4.8 nurses per thousand standard capita	(2) Ratio of beds per thousand standard capita	(3) Utilization (hospital days)	(1) Ratio of 4.8 nurses per thousand standard capita	(2) Ratio of beds per thousand standard capita	(3) Utilization (hospital days)	(1) Ratio of 4.8 nurses per thousand standard capita	(2) Ratio of beds per thousand standard capita	(3) Utilization (hospital days)	(1) Ratio of 4.8 nurses per thousand standard capita	(2) Ratio of beds per thousand standard capita	(3) Utilization (hospital days)
^"Calibrated" demand^	40,090	40,331	39,366	44,045	43,950	42,886	50,069	50,231	47,672	53,150	54,722	49,714
Total demand		39,347	38,406		42,878	41,840		49,006	46,509		53,387	48,501
Demand in hospital inpatients		22,539	21,463		24,459	23,300		28,417	25,937		31,246	26,778
Demand in outpatients		9,545	9,680		10,651	10,772		12,115	12,098		13,193	12,775
Demand in community		7,263	7,263		7,768	7,768		8,474	8,474		8,948	8,948

"Calibrated" demand for nurses working in places not included in the models (additional 2.5 % at each point in time)

(1) Demand projection based on ratio of 4.8 nurses per thousand standard capita, according to the average ratio of registered nurses employed in the population from 2009-2011

(2) Demand projection based on the model of a fixed ratio of hospital beds per thousand standard capita in the population for the inpatient departments (2.1 beds in general hospitals, 0.45 in psychiatric hospitals per thousand standard capita in addition to long-term nursing beds, per Stessman committee), demand projections for outpatient departments and demand projections for registered nurses in the community

(3) Demand projection consists of the demand projection based on the model of utilization of the health system (hospital days) for inpatient departments, the demand projection for outpatient departments, and the demand for registered nurses in the community

- Demand projections for Model A – ratio of nurses to the population (4.8 nurses per 1,000 standard capita): It is expected that in 2030, there will be a demand for 53,150 nurses
- Demand projections for Model B – ratio of beds per thousand capita: Based on the scenario of 2.1 general hospital beds and 0.45 psychiatric beds per thousand standard capita, plus long-term beds based on the Stessman Committee report, it is expected that in 2030 the demand for nurses will be 31,246 in general hospital wards, 13,193 in outpatient hospital units, and 8,948 in the community. With an additional 2.5 % for nurses working in places not included in the model, the demand for nurses will be 54,722.
- Demand projections for Model C – based on utilization (number of projected hospital days): In 2030, the demand for nurses in inpatient departments in hospitals is expected to be 26,778; 12,775 in outpatients and 8,948 in the community. With an additional 2.5 for nurses working in places not included in the model, the demand for nurses will be 49,714.

The table shows that the total demand for nurses in 2030, based on the projection model by utilization is lower than the projections using the models based on the ratio of nurses per capita and the ratio of beds to the population.

The difference can be explained by the fact that the ratio models assume an increase in the demand for nurses commensurate with the increase in the population, while taking account of the change in the composition of the population (utilization of standard population). In contrast, the demand model based on utilization used a series of linear regression analyses to examine the association between utilization (hospital days) and the population increase and the changes in its composition between 2001 and 2010 and applied this association to the future. This model takes account of the limitations of the system and its infrastructures and the system's adjustment to the growing population and changes in its composition without an increase in the direct ratio of the number of nurses.

### Balance between demand projections and supply projections

**Fig. 4:**
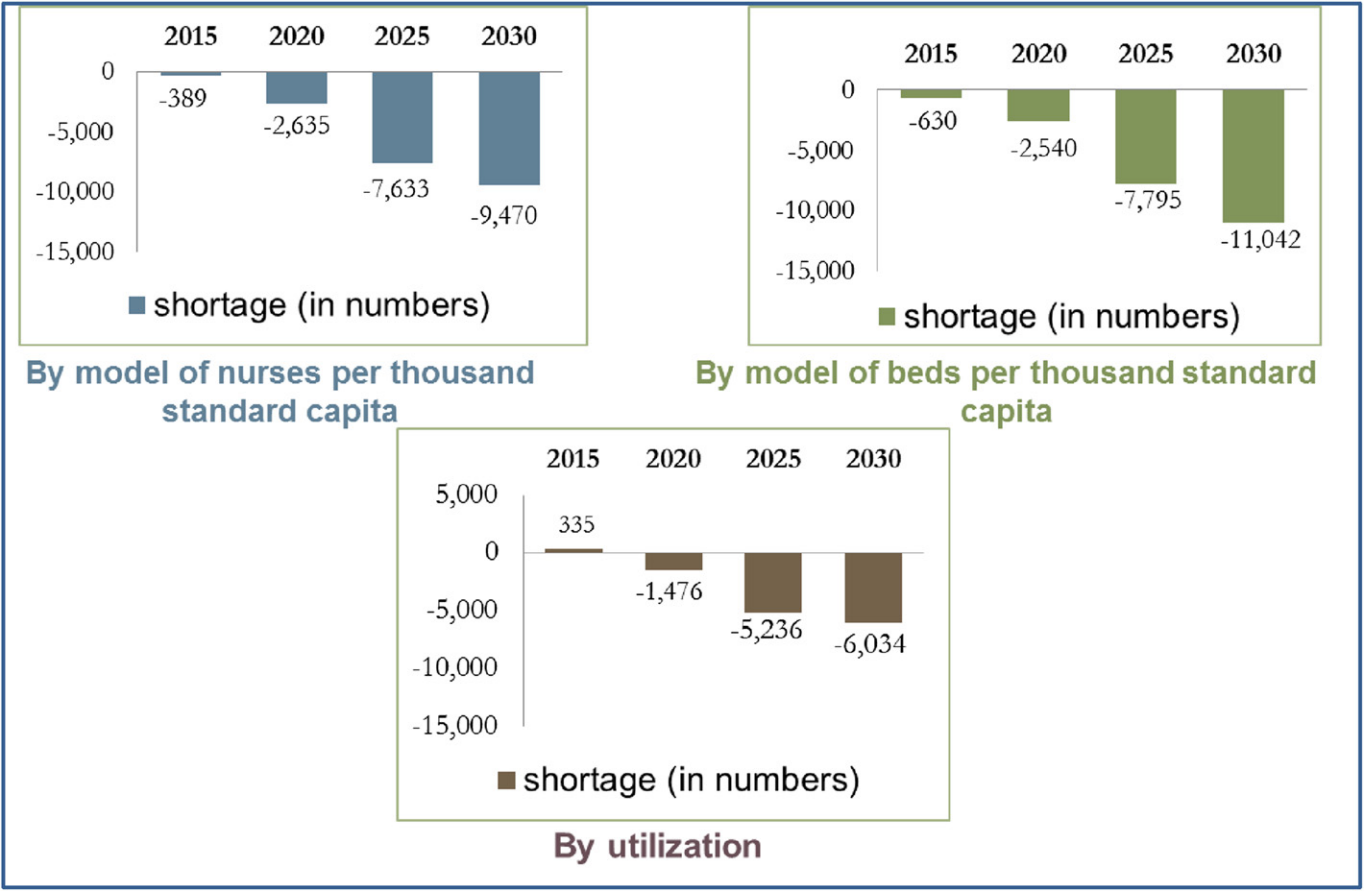
Gap between supply and demand projections – nurses in the workforce until age 65

**Fig. 5:**
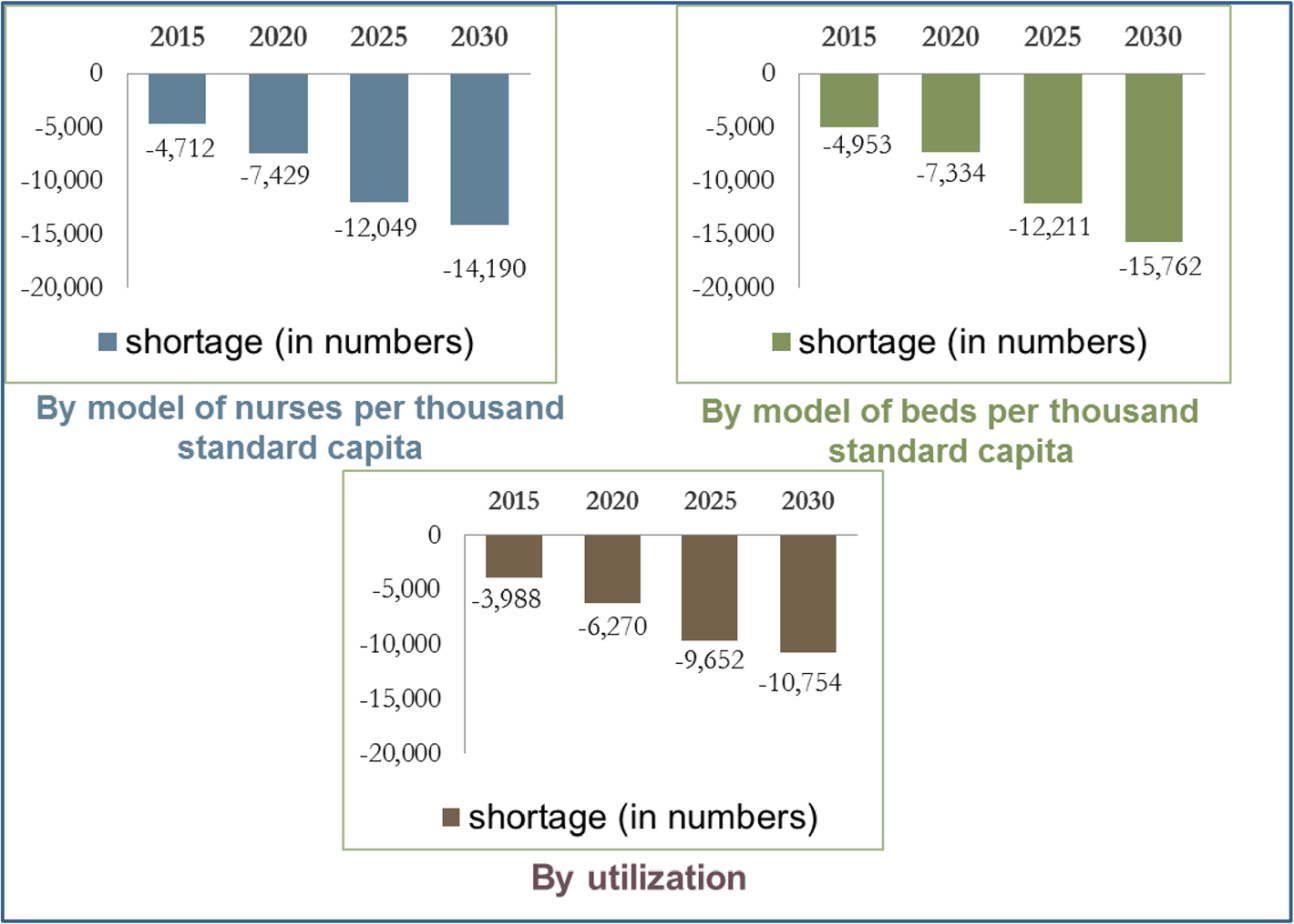
Gap between supply and demand projections – nurses in the workforce until age 60

When the balance between supply and demand projections was examined using the scenario where nurses are employed until age 60, the three models anticipated that there would already be a shortage of nurses in the short-term (2015). In the scenario where nurses remain in the workforce until age 65, two of the models show an anticipated balance between supply and demand or a moderate shortage of nurses in the short-term. However, all three models anticipate a considerable shortage in the long-term (2030). The extent of the shortage varies from one model to the next, but all three indicate that the anticipated shortage will be significantly affected by the age at which the nurses retire (see Figs. 4 and 5).

**Fig. 6:**
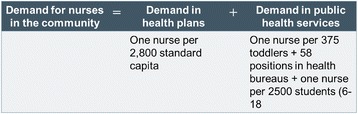
Formula for demand projections in the community

For example, an examination based on a fixed ratio of 4.8 nurses per thousand standard capita (Model A) reveals that already in 2015 there is expected to be a shortage of 4,700 nurses. The gap between supply and demand is expected to continue to grow and reach 14,190 in 2030. If the calculation is based on nurses remaining in the workforce until age 65, the gap will be smaller – a shortage of some 9,500 nurses in 2030.

When basing the calculation on a fixed ratio of hospital beds per thousand standard capita (Model B) with nurses retiring at age 60, we find that already in 2015 we can expect a shortage of some 4,850 nurses. The shortage is expected to increase until 2030, when there will be a shortage 15,760 nurses. If nurses remain in the workforce until 65, there will be a shortage of 630 nurses in the short-term (2015), which is expected to increase to 11,040 in the long-term (2030).

If the calculation is based on utilization (Model C), in 2015, we can expect a shortage of 4,000 nurses, if they retire at age 60. In 2030, the shortage will increase to 10,750. If nurses remain in the workforce until 65, we do not anticipate a shortage in 2015, but in 2030, there will be a shortage of some 6,000 nurses.

The study findings indicate that if nurses work until age 60, the annual percentage increase in the number required to cover the anticipated gap between supply and demand ranges from 2.3 % to 3 %, depending on the demand projection model. Similarly, if they work until age 65, the increase required will range from 1.5 % to 2.2 % annual, depending on the demand projection model.

## Discussion

The study examined three simple models that can be used by policymakers for demand projections for nurses. The advantage of first of them, which bases the demand projections on the ratio of nurses to thousand standard capita, is that, by using standard capita (by age, sex, and geographic area), it takes account of the change in the composition of the population.

The next two models also take account of population growth and the expected change in its demographic composition, but they also match them to the structure of the country's health system and the size of its infrastructures. The advantage of these models, particularly the one based on utilization, is that the projected demands do indeed reflect the increase in the workforce required to provide health services in the existing system. However, it must not be forgotten that these models are largely conservative. For example, despite the advantages of the model based on utilization, it is essentially based on the assumption that the system itself and the rules of the game will not change. Consequently, the projections are accurate only for a given situation (to a large extent this is also true for the model based on the ratio of beds per thousand capita). There might be changes that will affect these demand projections, e.g., a large increase in the number of hospital beds, a substantial increase in the training courses for nurses and recruitment of additional members of the workforce, new technology that will affect the essence of the nurses' work, changes in the standard ratio of nurses per bed in accordance with changes in the composition of patients in hospital departments, or the adoption of practical steps to substantially broaden the role of nurses in the community – and even a significant increase in the number of nurses required in the community. These or other possible changes in the health system would need to be entered into the demand projection equation to meet the new reality.

The literature on demand projections for the nursing workforce discusses the implications of other variables that were not included in the projection equations in the study, and that could affect the demand for nurses. The first of these is new medical technology, which increases the number of treatable medical conditions and could evidently significantly affect the role of nurses in the future [26, 27]. Although some claim that medical information technology will respond to the shortage of nurses by reducing the size of the workforce [28], we now know that not every technology reduces workload [29] and some may even create a greater demand for nursing staff than exists at present. The literature does assume there to be a direct connection between new technology, managed care, quality of care, and cost of care, but only few studies have examined this.

Another variable that could affect the demand for nurses is patient complexity. Nursing care involves caring for patients who suffer from a broad range of conditions, some of which may be life threatening. In the past twenty years, there has been an increase in the rate of chronic patients: Most patients age 65+ suffer from several chronic illnesses. Consequently, hospitalized patients of that age have more complex and severe conditions [30]. They need a high level of quality care that focuses on correct management of chronic illnesses and prevention of disability [31], creating a heavy workload for the nursing staff [32]. Despite this, it was found to be very difficult to quantify the complexity of care for such patients and to recommend the number of nurses required to deal with their complex medical problems [33, 34].

In addition to the difficulty quantifying the complexity of the patients' problems and care needs, there is no consensus among researchers about the probable changes in the future. One approach is that older age cohorts consume more health services [5]. The aging population has led not only to a substantial increase in the rate of chronic illnesses, but also to an increase in the number of complex patients who are in severe condition with several chronic illnesses, most of whom are aged 65+ [30]. These patients require more complex treatment and increase the workload on the medical and nursing staff in the hospitals. This trend is expected to continue. In contrast, following Fries [35, 36], it has been argued that caution is required with regard to the assumption that an increase in the complexity of patients can be expected in the developed Western world. According to this approach, thanks to preventive medicine, better medication and improved care, along with a healthier lifestyle, the onset of morbidity, chronic illness and disability will start later in life, while life expectancy will remain limited (despite increased life expectancy), leading to compression of morbidity for a shorter duration in a lifetime. Hence, the increase in the rate of the aging population is not necessarily associated with an increased burden on the health services (health expenditure or care provision). Furthermore, it is argued that the emphasis on aging population in discussion of the demand for medical workforce is not justified given that the impact of the aging process is expected to lessen due to a slower population growth. As a result the main implications will be on the mix of service providers rather than on their overall number [16, 37]. The inclusion of technological components and complex morbidity into the demand projection equation is a challenge that deserves attention in future research.

### Study limitations

The study has several limitations, the main ones of which are the difficulty of projecting possible change and the quality of data possessed by researchers. Every prospective demand study faces the difficulty of forecasting possible change. This is due to the many possible external influences on both supply and demand. External influences may be connected with policy change in health, such as system reforms, or to the introduction of new categories of health care professionals. The models suggested in the study remain valid if they are updated from time to time and the relevant changes are entered into the model equations.

Moreover, every prospective study is based on the data possessed by researchers, and these are true for the period of the study. The quality and accuracy of the data determine the quality of the projections. The more up-to-date the data system, the greater the chance that the projections will be more accurate. In our study, we attempted to draw data from the most up-to-date and reliable sources; nevertheless, some of the data, particularly those based on surveys, may not be entirely precise.

## Conclusions and implications for workforce planning policy

- The health system has been aware of the anticipated shortage of nurses for some time even if, until now, it has not had any agreed-upon data on the extent of the projected shortage. Therefore, action has already been taken and many resources are being invested in expanding the training courses for nurses and recruiting the workforce that will be required in the future. However, given the projections presented in the study regarding the predicted long-term (2030) shortage, it appears that the current efforts to increase the supply of nurses will not be sufficient and should be expanded. Thought should also be given to finding new ways to allocate additional resources in order to keep nurses in the workforce for many more years.
- Given the limited infrastructure and budget and the difficulty recruiting large numbers of nurses to the profession (not only in Israel), it may be that current new approaches are needed, such as introducing nurse assistants and/or other new categories of health care professionals in order to attract staff to the various levels of the nursing system. In other countries, new such support roles are being created to support registered nurses, creating a new mix of nursing workforce by adding professional levels that requires less training than of a registered nurse. The role of them is to assist and to take away some of the nurses' tasks, thereby reducing their workload and enabling them to use their time for a high standard of quality care of older, more complex patients with more complex technology than there used to be. This additional workforce would add to the nursing workforce quantitatively, but would also change the workforce mix.
- Studies that have examined the introduction of healthcare assistants and nurse assistance into hospital departments have found they have the potential to help with the difficulties arising from the shortage of registered nurses in those departments. However, the best way of doing so requires a clear job description and assessment of the optimal number of this workforce in relation to the nurses in the department [38–40].

The study, which provides information about the demand for nurses and the projected balance between supply and demand, should streamline the process of long-term strategic planning for the nursing workforce. The relatively simply models, based on available sources of information that can be easily revised, will make it possible to monitor and update projections regularly over time. The study findings can, furthermore, serve as the basis for projections to examine the balance between supply and demand and for workforce planning for other health service professions.

## Abbreviations

CBS: Central Bureau of Statistic

## Footnotes

1 The term nursing workforce includes professionals who have undergone different training programs to provide nursing care in the health service, including, registered nurses with different levels of training (graduates of 3-year training programs, academic programs, and retraining programs), practical nurses (since 2007, there has been no further training of practical nurses. However, according to a decision by the Ministry of Health of 30.4.2014 training of practical nurses will be renewed) ,and auxiliary staff, most of whom have undergone a short training program enabling them to work in hospital departments. ^2^Calculation of the demand projections for nurses in the community: In these projections, we applied the coefficient for the employment of nurses in the health plans in 2010 (weighted average of the ratio of nurses per health plan member) and coefficient for the employment of nurses in the public health system (mother and child clinics and student health), to the standard future population (per capita). The formula for demand projections in the community is as follows (Fig. 6):
